# Shared mobility services: an accessibility assessment from the perspective of people with disabilities

**DOI:** 10.1186/s12544-022-00559-w

**Published:** 2022-07-25

**Authors:** Anne Goralzik, Alexandra König, Laura Alčiauskaitė, Tally Hatzakis

**Affiliations:** 1 German Aerospace Center (DLR), Institute of Transportation Systems, Lilienthalplatz 7, 38108 Braunschweig, Germany; 2 European Network on Independent Living (ENIL), rue de l’industrie 10, Brussels, 1000 Belgium; 3 Trilateral Research Limited (TRI), 2nd Floor Marine Point, Belview Port, Waterford, X91 W0XW Ireland

**Keywords:** Mobility barriers, Access needs, Disability, Novel mobility solutions, Urban mobility, Shared transport, Online survey, Cross-European study

## Abstract

**Introduction:**

Shared on-demand mobility services emerge at a fast pace, changing the landscape of public transport. However, shared mobility services are largely designed without considering the access needs of people with disabilities, putting these passengers at risk of exclusion. Recognising that accessibility is best addressed at the design stage and through direct participation of persons with disabilities, the objective of this study was to explore disabled users’ views on the following emerging shared mobility services: (a) ride pooling, (b) microtransit, (c) motorbike taxis, (d) robotaxis, (f) e-scooter sharing, and (g) bike sharing.

**Methodolgy:**

Using an online mobility survey, we sampled disabled users’ (1) views on accessibility, (2) use intention, and (3) suggestions for improving accessibility. The results reflect the responses of 553 individuals with different types of disabilities from 21 European countries.

**Results:**

Projected accessibility and use intention were greatest for microtransit, robotaxis, and ride pooling across different disabilities. In contrast, motorbike taxis, e-scooter sharing, and bike sharing were viewed as least accessible and least attractive to use, especially by persons with physical, visual, and multiple disabilities. Despite differences in projected accessibility, none of the shared mobility services would fulfil the access needs of disabled persons in their current form. Suggestions for increasing the accessibility of these services included (a) an ondemand door-to-door service, (b) an accessible booking app, (c) real-time travel information, and (d) the necessity of accommodating wheelchairs.

**Conclusions:**

Our findings highlight the need for improving both vehicles and service designs to cater for the access needs of persons with disabilities and provide policymakers with recommendations for the design of accessible mobility solutions.

## Introduction

The current mobility strategy of the European Union (EU, [13]) highlights the need to make mobility services accessible and safe for all passengers including persons with disabilities and access needs. A lack of accessibility of transport hinders people with different types of disabilities and health problems from using mobility services, affecting their quality of life. It restricts their travelling, limits leisure opportunities, and can reinforce poverty by restricting access to opportunities for education and employment [38]. This issue does not affect a small group of individuals; it potentially concerns the lives of one in four EU citizens over the age of 16 years who report some form of long-term limitation in usual activities [14]. Studies show that despite many efforts in transport policy, public transport services remain largely inaccessible for people with disabilities [5, 32, 34]. A lack in accessibility has been shown to result from lacking or inaccessible information, e.g., if stops are not announced [5, 7, 20], inaccessible infrastructure, such as missing lifts [20, 34], inappropriate behaviour of drivers [5], inaccessible vehicles [16, 20], or a general inadequacy of public transport services, e.g., due to long pre-ordering times for assistance [37].

With new shared transport services, such as ride-pooling or micromobility services, emerging increasingly fast, it is an opportune moment to design them accessible from their inception. Shared mobility services have been defined as “transportation modes that allow riders to share a ride to a common destination-include various forms of ridesharing (carpooling and vanpooling); ridesourcing (or transportation network companies (TNCs)); microtransit; and taxi sharing” [35, p.1]. The present paper adopts a broader view in line with [25] who define shared mobility as “the short-term access to shared vehicles according to the user’s needs and convenience”. Thus, this paper includes mobility schemes in the definition of shared mobility services that are based on shared two-wheeled vehicles, like e-scooter sharing and bike sharing. Regarding current usage patterns, studies show that these systems are mainly used for leisure but rarely for commuting trips [22]**,** which particularly applies to e-scooter sharing systems [12]. Shared mobility services have been related to various benefits for their users, among them convenience [25], flexibility [12], financial [25] and environmental benefits compared to individual car use [21, 25]. However, several studies report concerns and challenges with respect to shared mobility services. For example, shared two-wheelers like e-scooters are often associated with conflicts with pedestrians or cyclists and a lack of safety [12]. Also, shared on-demand services are often more expensive than scheduled public transport [21] and therefore might exclude some user groups. Based on an equity assessment of emerging transportation systems, [16] conclude that the accessibility of shared mobility services is unequally distributed among different user groups. However, while this study considered different demographic groups, people with disabilities were not included.

To date, little is known about how people with disabilities use emerging shared mobility services [11]. First studies indicate heterogeneous usage patterns. For example, a national travel survey in the United States showed that people with disabilities use app-based ride-hailing services less often than other users, which points to existing barriers, like inaccessible apps and vehicles [9]. In contrast, a mobility survey in New York City revealed that travellers with disabilities are more likely to use on-demand ride-hailing services than a car or public transport [21].

To improve the accessibility of shared mobility services, it is crucial to understand users’ requirements. Research suggests that the type and severity of disability are important factors determining travel behaviour and user attitudes towards mobility services [8]. For example, for users with intellectual disabilities fear of technology plays a crucial role in their unwillingness to travel in autonomous vehicles, whereas increased familiarity with technology as well as a sense of control over technology increase their acceptance [3].

With regard to specific shared mobility schemes, studies point to considerable accessibility issues for people with disabilities [34]. For commercial ride-pooling services, like Uber, a study by [34] revealed several accessibility challenges, among them non-step-free access to vehicles, problems with wheelchair access and storage, and negative societal attitudes. Another study reported that major barriers to using campus shuttles are the hostility of drivers and physical inaccessibility of the vehicles [29].

With regard to autonomous vehicles, like robotaxis, [11] see a high potential for improved accessibility for the elderly and people with disabilities. However, other studies report concerns of people with physical [2, 10, 18] and visual disabilities [4, 33] with regard to autonomous vehicles, among them concerns over safety and affordability.

Regarding shared two-wheelers, like e-scooter sharing and bike sharing, research on accessibility for people with disabilities is still scarce [11]. However, there are first ideas to increase physical access, such as including tricycles in the bike-sharing fleet and offering e-scooters with seats [11].

To conclude, some studies expect benefits of shared mobility services for independent travel of people with disabilities [19, 28, 30], whereas others point to of accessibility issues of these services [6, 27]. There is some evidence about disabled users’ needs and preferences with regard to shared mobility systems; however, most of it is small-scale, localised, and not EU-based. Hence, it is difficult for such research to support EU policymakers in designing appropriate regulatory frameworks as well as social and educational strategies for creating the best possible conditions for bringing forward systemic change in the transport sector.

To address this research gap, the objective of this study was to explore how individuals with different types of disabilities from across Europe prospectively assess emerging shared mobility services, among them ride pooling, microtransit, motorbike taxis, robotaxis, e-scooter sharing, and bike sharing. The following research questions were investigated:

- How accessible are shared mobility services projected to be?
- Does projected accessibility of these services differ between people with different types of disabilities?
- What trip purposes would people with disabilities use these services for?
- What measures do people with disabilities suggest to increase the accessibility of these services?

These research questions were addressed by an online mobility survey which invited the participation of people with disabilities from all over Europe. The present study amplifies current knowledge of user attitudes towards shared mobility services by addressing an understudied user group. Based on the results, implications for transport policy are derived that aim to make emerging shared mobility services more accessible for people with disabilities.

## Methodology

### Survey design

The present study used data from an online mobility survey that addressed people with disabilities [17]. The survey included different sets of closed-ended and open-ended items that cover eight mobility-related topics, including the accessibility assessment and use intention of emerging mobility services.

A total of nine emerging mobility services were presented in the survey, six of which were shared mobility services and fell within the scope of this study: (a) ride pooling, (b) microtransit, (c) motorbike taxis, (d) robotaxis, (e) e-scooter sharing, and (f) bike sharing. Ride pooling, microransit, and robotaxis describe services that may transport more than one passenger at a time. Conversely, motorbike taxis, e-scooter, and bike sharing schemes are designed to be used sequentially by one passenger at a time. Each shared mobility service was introduced by a short text describing its operation, booking, and a potential use case. Three questions were presented to record the accessibility of the respective mobility service.

First, respondents were asked to evaluate a service’s impact on the quality of their journey if that service could be made available. Expected journey impact was measured on five travel-related dimensions: autonomy (“If we could make this system accessible, would it make your journey more independent?”), travel time (“…make your journey faster?”), convenience (“…make your journey easier?"), comfort (“…make your journey nicer?”), and safety (“…make you feel safe?”). These five dimensions form part of the Mobility Divide Index (MDI) developed by [1] which measures the accessibility level of public transport for people with disabilities. Additionally, respondents were asked if the service would increase their motivation to travel (“…make you want to travel more?”) on a sixth dimension. Respondents indicated their level of agreement with these questions on a 5-point Likert scale with the response options “no” (1), “not a lot” (2), “don’t know” (3), “quite a bit” (4), and “yes” (5).

Second, respondents’ intention to use the shared mobility services for different trip purposes (education, commuting, shopping, scheduled appointments, leisure) was registered. Answers were recorded on a 3-point rating scale (“yes”, “maybe”, “no”). Additionally, there was the option to state “not applicable”.

Third, an open-ended question was aimed at eliciting suggestions for increasing the accessibility of the respective service (“What would you need to make this system work for you?”). Responses were given via text input.

The survey was developed in close collaboration with local disability user groups in seven European cities (Bologna and Cagliari, Italy; Brussels, Belgium; Lisbon, Portugal; Sofia, Bulgaria; Stockholm, Sweden; Zagreb, Croatia). It was first drafted in English and then translated into 14 European languages (Bulgarian, Croatian, Dutch, French, German, Greek, Italian, Lithuanian, Polish, Portuguese, Romanian, Russian, Spanish, and Swedish).

### Procedure

The survey was created and conducted using the online software SoSci Survey [24]. The survey could be completed either by individuals with disabilities themselves or, if they were unable to do so, by another person answering on their behalf. Before the survey, respondents were informed about its purpose and gave their consent for their participation and the use of their anonymous responses for research and publication purposes. Also, a question about whether the respondent faced a disability was included at this stage to filter out non-disabled respondents.

The survey took, on average, 20 to 30 min to complete. All questions were mandatory for respondents. To minimise survey completion time, a random subset of two out of the six shared mobility services were presented to each respondent. The survey was distributed using a snowball system using three multiplicator routes: (1) local disability user groups in the seven European cities, (2) local, national, and international organisations working with people with disabilities, and (3) social media.

A research ethics statement covering research design guiding principles and practices, data management policies in line with the EU General Data Protection Regulation, and informed consent procedures was reviewed and approved by the Ethics Advisory Officer of the project within which this study was conducted.

This paper presents data collected between 1 November 2020 and 12 February 2021.

### Sample

**Fig. 1:**
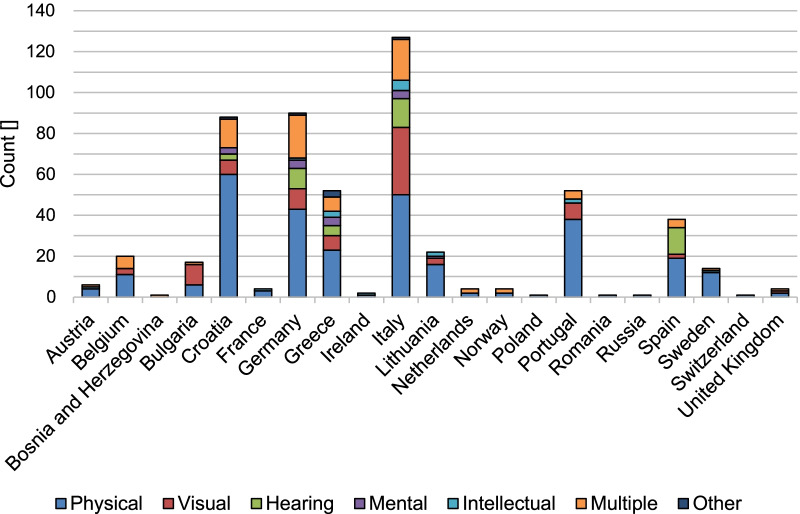
Completed surveys per country of residence split by type of disability (*n* = 549; note: data on country and type of disability were unusable and therefore discarded for *n* = 2 surveys each; respondents whose disabilities did not fit into any of the pre-defined categories were assigned to the category “Other”)

Out of a total of 872 submitted surveys, 319 surveys were excluded from analysis because they were either completed by non-disabled respondents (*n* = 312), by respondents from non-European countries (*n* = 3), or because they contained wrongly recorded data (*n* = 4). The final sample included 553 surveys in all 15 languages, completed by individuals from 21 European countries. Figure 1 shows the number of completed surveys per country of residence and by type of disability. What stands out in this figure is the uneven distribution of completed surveys between countries. Almost three quarter of the surveys were filled in by residents of only five countries, i.e. Croatia, Germany, Greece, Italy, and Portugal. Also, the proportions of the different disabilities stated in the surveys varied to a large degree between countries. For instance, in the five countries with the highest turnout the share of respondents with a physical disability varied between 39.4% (Italy) and 73.1% (Portugal).

Most surveys were completed by individuals with disabilities themselves (87.7%) and the rest by another person answering on their behalf. There were slightly more male respondents (51.4%) than female respondents (45.8%); the remaining respondents identified as diverse or did not state their gender. A breakdown of the sample by type of disability, age, and employment status is shown in Table 1. Respondents with physical disabilities constituted more than half of the sample, whereas respondents with mental health issues or intellectual disabilities were only marginally represented. Employment status varied across disabilities. The share of respondents working or studying was highest among those with visual or hearing impairments and lowest among those with intellectual or multiple disabilities. While approximately one fifth of respondents with visual, hearing, intellectual, and multiple disabilities were unemployed, unemployment was slightly lower in the group with physical disabilities and slightly higher in the group with mental health issues. The share of retired respondents was largest among those with multiple disabilities and lowest among those with hearing impairments. Other types of occupation, such as community service, were relatively rare across disabilities except for respondents with intellectual disabilities who stated other types of occupation just as frequently as work and study combined.

**Table 1:** Age and employment status in the total sample and by type of disability

Type of disability	*n*^a^	(%)	Age [years]	Employment status [%]^b^				
			*M* (*SD*)	Unempl.^c^	Work	Study	Retired	Other
Total	553	100.0	46.4 (15.7)	15.9	41.2	8.0	28.0	6.9
Physical	297	53.7	48.8 (15.8)	12.8	39.4	7.1	34.0	6.7
Visual	85	15.4	39.7 (13.2)	18.8	56.5	11.8	10.6	2.4
Hearing	45	8.1	42.5 (12.8)	20.0	60.0	8.9	6.7	4.4
Mental	16	2.9	49.3 (12.7)	25.0	43.8	0.0	25.0	6.3
Intellectual	17	3.1	37.0 (16.9)	17.6	23.5	11.8	11.8	35.3
Multiple^d^	85	15.4	47.8 (16.8)	20.0	28.2	7.1	36.5	8.2

^a^*n*: Eight respondents did not fit in any of the subsamples. Therefore, the number of respondents in the subsamples does not add up to the total *N*

^b^Due to rounding errors, employment status [%] does not add up to 100% in the subsamples ‘Visual’ and ‘Mental’, respectively

^c^Unempl.: Unemployed

^d^Multiple: More than one type of disability was stated

### Data processing and analysis

Responses to the closed-end questions, which recorded projected accessibility and use intention of the shared mobility services, were analysed descriptively. Relative frequencies of responses were calculated individually for each question and separately for each type of disability. Additionally, a mean score of the ratings across all travel-related dimensions was calculated for each shared mobility service to provide an overall measure of the projected accessibility of the service. Written suggestions for improving the mobility services were first translated from the local language of the respondents into English. They were then categorised with the software MAXQDA, using an inductive content analysis approach [26]. Due to the large variation in the number of completed surveys and in the distribution of disabilities between the countries (see Fig. 1), a comparison of results on the country-level would have carried the risk of biased conclusions. Therefore, the analyses pooled data from all countries.

## Results

### Projected accessibility

#### Projected accessibility by dimensions of journey quality

**Fig. 2:**
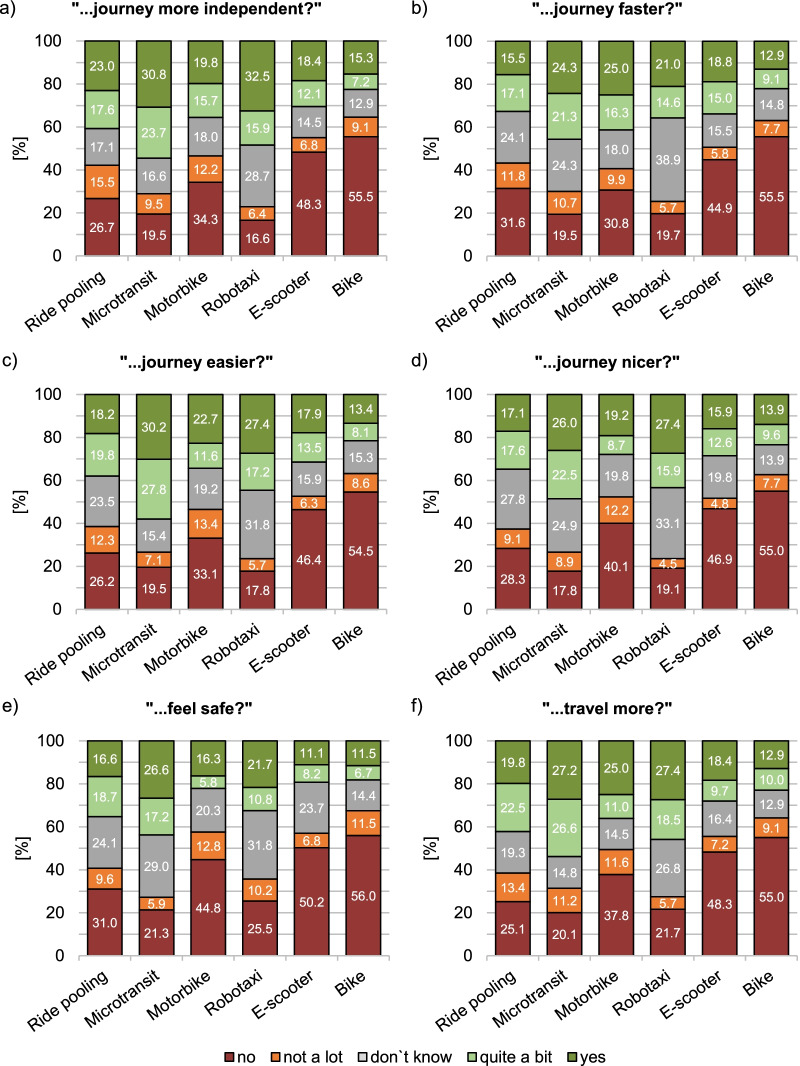
Assessment of journey quality across disabilities along the travel-related dimensions **a** autonomy, **b** travel time, **c** convenience, **d** comfort, **e** safety, and **f** motivation to travel; note: the size of subsamples differed between the mobility services; ride pooling: *n* = 187, microtransit: *n* = 169, motorbike taxi: *n* = 172, robotaxi: *n* = 157, e-scooter sharing: *n* = 207, bike sharing: *n* = 209)

This section presents respondents’ assessment of the accessibility of the shared mobility services along the six dimensions of journey quality. Figure 2 shows respondents’ expectations of how much their journey would improve regarding the six dimensions using the respective mobility services, pooled across different types of disabilities.

Ride pooling evoked mixed expectations concerning autonomy, travel time, convenience, comfort, safety, and motivation to travel. Here, the proportion of respondents who thought that this mobility service would improve their journey at least quite a bit across these dimensions (*M* = 37.3%) and who thought that it would not or not a lot (*M* = 40.1%) was similar.

Regarding microtransit, there were more respondents expecting their journey to improve at least to some degree on each of the journey dimensions than there were respondents thinking that their journey would not improve or would not improve much. Specifically, around half of the respondents (*M* = 50.7%) expected a higher degree of autonomy, convenience, and motivation to travel using microtransit.

Motorbike taxis received mixed ratings, although the share of respondents estimating that their journey would not or would not a lot be improved using this service (*M* = 48.8%) outweighed the share of respondents who thought it would (*M* = 32.8%). Around half of the respondents expected no or little improvement in journey quality using motorbike taxis except for travel time. For travel time, the share of positive ratings (∑ = 41.3%) and negative ratings (∑ = 41.7%) was almost equal.

Regarding robotaxis, a considerably higher proportion of respondents estimated this mobility service to positively impact their journey (*M* = 41.7%) than to negatively impact it (*M* = 26.4%). This was the case for all journey dimensions except for safety, where the relative frequencies of positive ratings (∑ = 32.5%) and negative ratings (∑ = 35.7%) were fairly similar. There was also a considerable proportion of respondents who were undecided if this service would positively affect their journey, ranging between 26.8% (motivation to travel) and 38.9% (travel time).

Using e-scooter sharing, more than half of the respondents (*M* = 53.8%) expected that it would not or would not much improve the quality of their journey on any of the journey dimensions. Among the remaining respondents, the share of those giving e-scooter sharing a clear positive rating (*M* = 17.9%) was similar to the share of undecided respondents (*M* = 16.4%) except for the safety dimension, where the share of undecided respondents (∑ = 23.7%) was a little more than double the share of clear positive respondents (∑ = 11.1%).

Bike sharing was judged as not having a positive impact on the different dimensions of the journey by nearly two-thirds of the respondents (*M* = 64.2%), with more than half ruling out any positive effect (*M* = 55.3%). The proportions of those with a clear positive assessment of bike sharing (*M* = 13.3%) and of those who were undecided (*M* = 14.0%) were about the same.

Summarising the pattern of responses shown in Fig. 2, ratings differed more between the shared mobility services than between the dimensions of journey quality. Even though responses were mixed, microtransit and the robotaxi received a larger share of positive than negative responses. Respondents were ambiguous about ride pooling and had the lowest expectations for an improved journey using e-scooter sharing or bike sharing.

#### Projected accessibility by disability

**Fig. 3:**
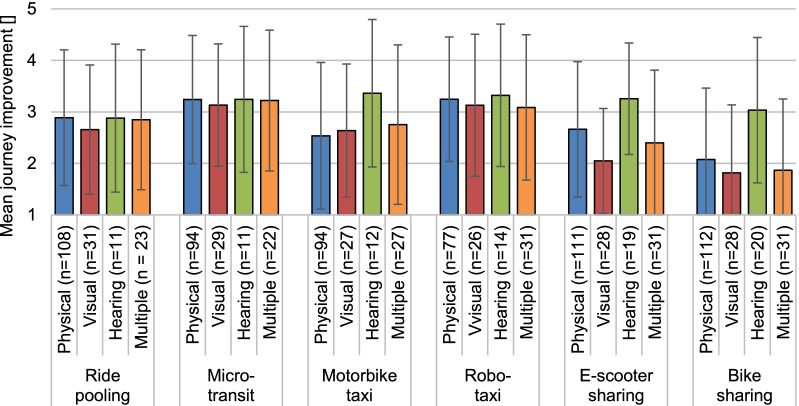
Projected overall journey improvement using the shared mobility services by type of disability (mean score with *SD* with 1 = very little improvement, 5 = very large improvement; note: the *n* in each column corresponds to approximately a third of the total *N* per type of disability. The different numbers of responses result from the randomized presentation of two out of six shared mobility services)

Because the dimensions of journey quality were rated very similarly for each mobility service, we used a mean score summarising the ratings to compare the projected overall journey improvement with a mobility service between different disabilities (see Fig. 3). However, we excluded mean scores of respondents with mental health issues or intellectual disabilities due to very small subsample sizes (*n* ≤ 8), as the generalisability of the respective results would be restricted.

As Fig. 3 shows, all four subsamples had ambiguous expectations towards an improvement in the overall journey using ride pooling (*M* = 2.9, *SD* = 1.3), microtransit (*M* = 3.2, *SD* = 1.2), and the robotaxi (*M* = 3.2, *SD* = 1.3). Also, the ratings of these three mobility services were fairly similar between respondents with physical (*M* = 3.1, *SD* = 1.3), visual (*M* = 3.0, *SD* = 1.3), hearing (*M* = 3.2, *SD* = 1.4), and multiple disabilities (*M* = 3.1, *SD* = 1.4). Variation in expected journey improvement between respondents with different disabilities were notable for the motorbike taxi, e-scooter sharing, and bike sharing. While respondents with hearing impairments were undecided whether these mobility services would improve their journey (*M* = 3.2, *SD* = 1.3), expectations of respondents with physical (*M* = 2.4, *SD* = 1.4), visual (*M* = 2.2, *SD* = 1.3), or multiple disabilities (*M* = 2.3, *SD* = 1.5) were more negative. Especially using e-scooter sharing and bike sharing, respondents with visual (*M* = 1.9, *SD* = 1.2) and multiple disabilities (*M* = 2.1, *SD* = 1.4) expected little improvement in their journey. Respondents with physical impairments were slightly more optimistic about e-scooter sharing (*M* = 2.7, *SD* = 1.3) than bike sharing (*M* = 2.1, *SD* = 1.4), which they rated similarly low as respondents with visual and multiple disabilities.

To summarise, regardless of disability ride pooling, microtransit, and the robotaxi received a medium accessibility score, reflecting an ambiguity towards these mobility services. Respondents with hearing impairments displayed this ambiguity towards all shared mobility services considered. In contrast, accessibility scores for the motorbike taxi, e-scooter sharing, and bike sharing were lowest in the subsamples with physical, visual, and multiple disabilities.

### Use intention

**Fig. 4:**
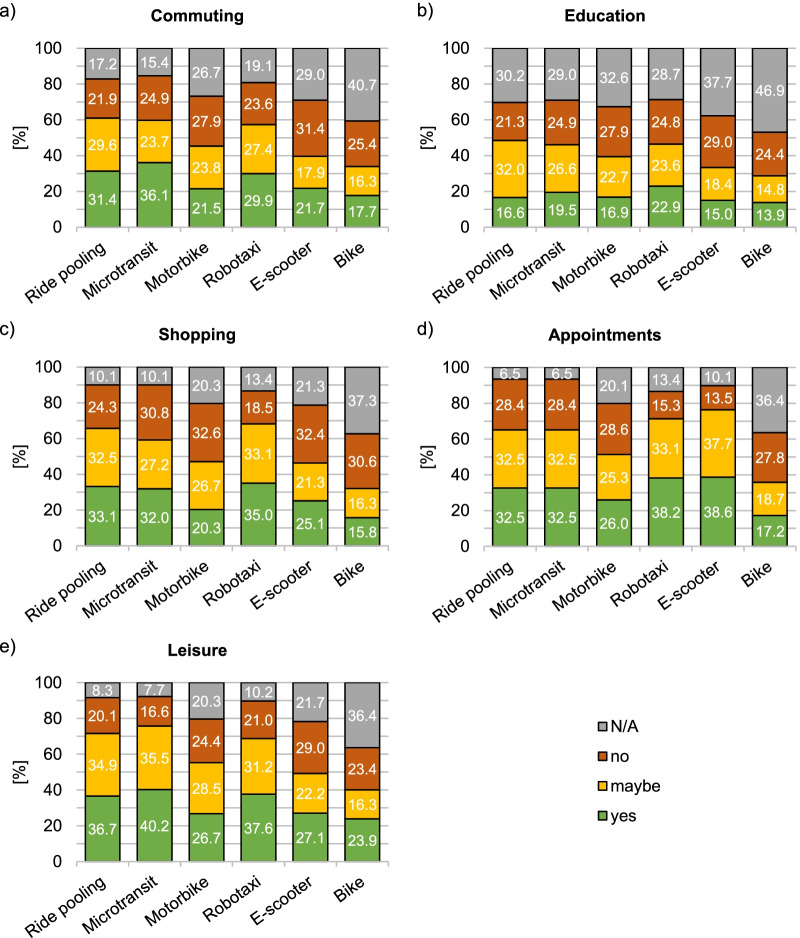
Intention to use the shared mobility services for the different trip purposes **a** commuting, **b** education, **c** shopping, **d** appointments, and **e** leisure across disabilities (N/A: not applicable)

This section presents respondents’ willingness to use the six shared mobility services for different trip purposes. Use intention is displayed in Fig. 4 for the purposes of (a) commuting between home and work, (b) educational purposes, (c) going shopping, (d) going to scheduled appointments, and (e) leisure activities across different disabilities.

Irrespective of the trip purpose, respondents’ willingness to use the shared mobility services varied widely (see Fig. 4). Use intention, defined by both “yes” and “maybe” responses, was most pronounced for ride pooling (*M* = 62.4%), microtransit (*M* = 61.2%), and the robotaxi (*M* = 62.4%). Here, at least half of the respondents expressed an openness to use these services across trip purposes, except for education-related trips for which openness was lower (∑ = 40.5%). Furthermore, nearly half of the respondents would be open to use motorbike taxis (*M* = 47.7%) and e-scooters (*M* = 49.0%). Finally, less than half of the respondents would consider bike sharing for any type of trip (*M* = 34.2%). Among those respondents who indicated a general openness to use a shared mobility service, the proportions of those clearly in favour and those cautiously open tended to be balanced across trip purposes.

The degree of (un-)willingness to use a given service was comparable between the trip purposes of commuting, going shopping, and leisure (see Fig. 4). Imagining going to an appointment increased the share of respondents who were at least open to using the robotaxi (∑ = 71.3%) or e-scooter sharing (∑ = 76.3%) compared to using these services for other trip purposes (*M*_robotaxi_ = 60.2%, *M*_e-scooter_ = 42.2%). Making trips for educational purposes was least attractive with any of the mobility services, with less than one in five respondents indicating a clear willingness to do so (*M* = 17.5%).

There was considerable variation in the extent to which trip purposes were rated as applicable at all. Educational trips were rated as least applicable among all trip types, with the share of “non-applicable” ratings ranging between 28.7% and 46.9%, followed by commuting trips (15.4–40.7% of “non-applicable” ratings). Of the examined shared mobility services, bike sharing received the highest proportion of “non-applicable” ratings for any trip purpose (36.4–46.9%).

In conclusion, ride pooling, microtransit, and the robotaxi were the most attractive mobility services for different types of trips. The intention to use a given mobility service was less related to the specific type of trip than to the service itself.

### Users’ suggestions for improving accessibility

In the following, respondents’ suggestions for improving the accessibility of the six mobility services are presented based on the answers to the open question “What would you need to make this system work for you?”.

With regard to ride pooling concepts, the suggestions implied that prospective users would wish them to be highly flexible and available on-demand without the need for prior reservations. Further suggestions pointed to the need for a door-to-door service that picks up passengers at any place. Another suggestion addressed real-time localisation of the vehicle to facilitate its tracking.

For microtransit, the suggestions dealt with the need for a reliable and punctual service. As with ride pooling, respondents suggested real-time tracking of the vehicle. The need for wheelchair access and voice notification of stops were suggested as well. Another frequently addressed issue was the affordability of the service.

Regarding the motorbike taxi, the suggestions included adjusted vehicles that are able to transport wheelchairs. Some respondents suggested motorbike taxis use four-wheelers rather than two-wheelers. Further suggestions pointed to the need for making the service affordable and to cost less than a conventional taxi.

With regard to robotaxis, the suggestions emphasised the necessity for ensuring safety, e.g., by providing a separate infrastructure for autonomous vehicles to avoid sharing space with human drivers. Furthermore, the need for both visual and auditive information about the vehicle status was emphasised. Adding to this, respondents suggested a smart notification system to identify the pick-up stop and the vehicle.

To increase the accessibility of e-scooter sharing schemes, respondents requested adjusted vehicles that balance themselves, e.g., by the help of additional wheels or by providing tandems with a driver who rides the e-scooter. In addition, some respondents with visual impairments suggested e-scooters to be self-driving to facilitate mobility without the need for human assistance. Further recommendations pointed to the need for a separate infrastructure, e.g., dedicated lanes.

Most of the suggestions concerning bike sharing related to the need for bikes to be adjustable, e.g., by providing shared handbikes and with electric support. Suggestions also included vehicles that support balance, e.g., tricycles or four-wheelers, but also tandems. Individuals with visual and hearing impairments suggested equipping the bike with a device that observes the environment and issues warnings.

Several suggestions were named across all shared mobility services. Among them were a reservation system that would not require mobile internet access, a door-to-door service, affordability, and safety of the services. Furthermore, several statements pointed to the wish of not sharing a vehicle during an individual ride, among them robotaxis, microtransit, and ride-pooling schemes, to avoid contact with other travellers. For all mobility services, a fully accessible booking app was requested by the respondents regardless of their disability.

## Discussion

This section covers four topics: (a) summary and reflection of the results, (b) limitations of this study and further research, and (c) implications for transport policy. Summarising the results, projected accessibility of all shared mobility services varied to a large degree between respondents, indicating that none of the services in its current format would entirely fulfil the divergent access needs of persons with physical, sensory, or cognitive impairments. Microtransit and the robotaxi raised the highest expectations for an improved journey, followed by ride-pooling schemes across different disabilities. There is a need for these services to be versatile by providing on-demand rides from door to door, accessible real-time status updates about the vehicle and the trip, as well as space to accommodate wheelchairs.

The ambiguity of a large share of respondents towards the robotaxi is in line with the novelty of the service. It implies that while persons with access needs may be open to travelling in an autonomous vehicle, as previously shown [11, 19], more information about its service design and operation are needed. Respondents’ requirements highlight disabled travellers’ need for reassurance that autonomous transport is safe for them to use, confirming recent studies [4, 33]. However, the requirements were not specific enough to draw conclusions about the aspects feeding into the safety perception of people with disabilities (e.g., predictability, controllability, error tolerance, or security) and if these aspects differ from other groups of travellers. Therefore, further research with respect to the specific safety needs of travellers with disabilities when using autonomous transport is recommended.

There is a lack of studies addressing the accessibility of two-wheeled shared mobility services from the perspective of people with disabilities [11]. This study contributes to filling this research gap, demonstrating that the perceived accessibility of motorbike taxis, e-scooter sharing, and bike sharing was low. This was especially the case for people with physical, visual, and multiple disabilities. The very design of these mobility services and the requirements they impose on the user may explain this finding. Unless retrofitted, these mobility solutions require active participation of the user by using the legs to get on and off the vehicle and maintaining balance. In the case of conventional two-wheeled e-scooters and bicycles, users operate the vehicle themselves, which requires them to be able to perceive and respond to the traffic environment as well as to use their legs for standing or pedalling. Whereas hearing-impaired users appeared somewhat less affected by a greater degree of participation using a two-wheeler, users with physical, visual, or multiple disabilities are at risk of exclusion from using these mobility solutions due to a mismatch between vehicles’ design and users’ requirements. Reflecting the access needs of these user groups, suggestions for these mobility services included vehicles that are compatible with wheelchairs, self-balancing, and provide electric support up to being entirely self-driving. These design suggestions add to the ideas presented by [11].

Our results suggest that the accessibility of shared mobility services depends crucially on the degree of participation required of the user. The more active the role of users with disabilities is, the lower can the acceptability and use intention be expected to be.

Consistent with this, the intention to use ride pooling, microtransit, or robotaxi services tended to be higher than for motorbike taxis, e-scooter sharing, or bike sharing. However, there was no clear indication about users’ preference for a shared mobility service depending on the purpose of trip, implying that the purpose of the trip itself is less critical to usage than the perceived accessibility of the service. Additionally, a considerable share of trips was categorised as “non-applicable”, which the present study cannot fully explain. Non-applicable trips may have resulted from a lack of necessity for a trip, e.g., persons who work may have no need to go to an educational facility, or the assessment that the mobility service is unsuitable because of the disability.

The findings reveal some degree of unwillingness to share a ride with fellow passengers when using robotaxis, microtransit, and ride-pooling schemes. A possible explanation might lie in the timing of the survey during the COVID-19 pandemic, which led to a reduction in trips with public transport due to the fear of an infection [15, 23]. Health concerns might also decrease the acceptance of shared mobility schemes [36], with early evidence pointing to a reduction in trips using taxis and ride pooling by people with disabilities [31].

While this study provides insights into the accessibility assessment, use intention, and requirements of people with disabilities regarding shared mobility services, it can only speculate about the underlying motivation for the responses obtained. For an enhanced understanding of disabled people’s attitudes and usage barriers, we recommend further research using interviews and behavioural observation.

Due to the prospective nature of this study, the respondents did not experience the shared mobility services on the road. It would therefore be valuable for future research to assess a posteriori experiences and compare them with a priori expectations. Such a comparison could, for instance, reveal the need for improving a mobility service if expectations exceed experiences or, alternatively, for increasing awareness about a mobility service if the actual experience turns out better than expected.

This survey reached a considerable number of people with disabilities from a multitude of European countries. However, people with mental health issues and intellectual impairments were underrepresented, potentially reflecting a self-selection bias. Thus, their views may not have been sampled appropriately. Targeted efforts should be made in future studies to reach out to these groups and in designing accessible survey material, e.g., by presenting information in easy read. While we obtained a broad European database, we primarily strived to maximise responses per disability and not per country. Therefore, turnout varied between countries and was low for most countries, rendering a comparison of results on the country-level futile. Given that the country of residence may play a role in one’s attitudes towards mobility solutions, it would be valuable to increase the representation of each country in future research to allow cross-country comparisons.

Finally, in addition to people with long-standing disabilities, other groups may be expected to face mobility barriers. For this reason, a second wave of the online survey will be rolled out, addressing people with milder mobility impairments, such as temporary impairments due to injuries or pregnancy. The data of the second survey will be compared with the sample of people with disabilities to broaden the view on the accessibility of emerging shared mobility services.

Overall, our results indicate the following design and policy considerations to be addressed:

• Redesign shared mobility services based on the access needs of users facing mobility barriers.
• Invest in the accessibility of the transport infrastructure, like dedicated lanes for micromobility solutions.
• Include accessibility experts and users with disabilities in the development of vehicles, mobility infrastructure, and mobility services.

## Conclusion

The objective of this paper was to investigate the projected accessibility of emerging shared mobility services from the perspective of persons with disabilities from across Europe. We analysed the expected impact of these mobility services on journey quality, the prospective use of these services, and accessibility requirements. Our main findings underscore the assumption that emerging shared mobility services are currently not designed and operated in a way that ensures equal access for people with disabilities. Despite considerable variation in projected accessibility, microtransit, robotaxis, and ride pooling were identified as having the greatest potential for improving mobility across disabilities. In contrast, motorbike taxis, e-scooter sharing, and bike sharing were viewed as much less accessible, with considerable differences between disabilities. Our study provides ideas for improving the accessibility of shared mobility services and vehicles as considerations for policymakers while demonstrating the value of listening to the needs of users with disabilities, who are the experts of their own access needs.

## Data Availability

Data and materials are available upon request.

## Abbreviations

EU: European union
MDI: Mobility divide index
